# Altered *Mycobacterium tuberculosis* Cell Wall Metabolism and Physiology Associated With RpoB Mutation H526D

**DOI:** 10.3389/fmicb.2018.00494

**Published:** 2018-03-19

**Authors:** Victoria L. Campodónico, Dalin Rifat, Yu-Min Chuang, Thomas R. Ioerger, Petros C. Karakousis

**Affiliations:** ^1^Department of Medicine, The Johns Hopkins University School of Medicine, Baltimore, MD, United States; ^2^Department of Computer Science and Engineering, Texas A&M University, College Station, TX, United States; ^3^Department of International Health, Johns Hopkins Bloomberg School of Public Health, Baltimore, MD, United States

**Keywords:** *Mycobacterium tuberculosis*, *rpoB* mutation, rifampin, cell wall, vancomycin

## Abstract

**Background:** *Mycobacterium tuberculosis* (Mtb) *rpoB* mutations are associated with global metabolic remodeling. However, the net effects of *rpoB* mutations on Mtb physiology, metabolism and function are not completely understood. Based on previous work, we hypothesized that changes in the expression of cell wall molecules in Mtb mutant RpoB 526D lead to changes in cell wall permeability and to altered resistance to environmental stresses and drugs.

**Methods:** The phenotypes of a fully drug-susceptible clinical strain of Mtb and its paired rifampin-monoresistant, RpoB H526D mutant progeny strain were compared.

**Results:** The *rpoB* mutant showed altered colony morphology, bacillary length and cell wall thickness, which were associated with increased cell wall permeability and susceptibility to the cell wall detergent sodium dodecyl sulfate (SDS) after exposure to nutrient starvation. Relative to the isogenic rifampin-susceptible strain, the RpoB H526D mutant showed altered bacterial cellular metabolic activity and an eightfold increase in susceptibility to the cell-wall acting drug vancomycin.

**Conclusion:** Our data suggest that RpoB mutation H526D is associated with altered cell wall physiology and resistance to cell wall-related stress. These findings are expected to contribute to an improved understanding of the pathogenesis of drug-resistant *M. tuberculosis* infections.

## Introduction

Tuberculosis (TB) remains a major threat to global public health (Lawn and Zumla, 2011), with an estimated 1.7 billion people infected with *Mycobacterium tuberculosis* (Mtb) globally (WHO, 2017). Rifampin is one of the two most effective anti-TB drugs (Gagneux et al., 2006). Although rifampin monoresistance accounts for only ∼18% of new drug-resistant cases (WHO, 2017), resistance to rifampin is considered a surrogate marker for multidrug-resistant (MDR) TB, i.e., TB resistant to the two first-line antibiotics, isoniazid and rifampin. MDR TB is a major barrier to successful TB control (Dheda et al., 2017) due to the inferior efficacy, and increased length, toxicity and cost of MDR-TB treatment (Manjelievskaia et al., 2016).

Rifampin resistance is conferred by chromosomal mutations in the *rpoB* gene encoding the β subunit of the RNA polymerase (RpoB) (Alifano et al., 2015). Most rifampin-resistance-conferring mutations in Mtb clinical isolates are due to amino acid changes at codons 531, 526, 522, and 513 of RpoB (based on the *Escherichia coli* annotation). Rifampin-resistance-conferring *rpoB* mutations have effects on mycobacterial transcription (Jin and Gross, 1989; Cai et al., 2017), and, consequently, the fitness of the organism (Reynolds, 2000), which varies depending on the specific *rpoB* mutation, the genetic background of the antibiotic-resistant mutant and between different bacterial species (Gagneux et al., 2006; Alifano et al., 2015). However, the global effects of *rpoB* mutations on *Mtb* physiology, metabolism and function are not well characterized. The impact of *rpoB* mutations on Mtb physiology has been studied previously using high-throughput approaches. For instance, transcriptomic and proteomic analysis of Mtb clinical isolates containing *rpoB* mutations demonstrated upregulation of polyketide synthases, which are enzymes involved in the biosynthesis of phthiocerol dimycocerosate (PDIM) (Bisson et al., 2012). More recently, lipidomic profiling of *rpoB* mutant Mtb showed altered concentrations of mycobactin siderophores and acylated sulfoglycolipids (Lahiri et al., 2016), while metabolomics revealed decreased synthesis of various branched-chain fatty acids (du Preez and Loots du, 2012). These cell wall-associated lipids, including PDIM, mycobactin, sulfoglycolipids, and fatty acids, have been implicated in cell wall permeability and virulence (Camacho et al., 2001; Converse et al., 2003; Yeruva et al., 2006; Gebhardt et al., 2007; Gilmore et al., 2012; Reddy et al., 2013; Lahiri et al., 2016; Quigley et al., 2017).

We hypothesized that changes in the expression of these molecules in an Mtb rifampin-resistant strain containing the mutation RpoB 526D leads to changes in cell wall permeability, as well as altered resistance to environmental stresses and drugs.

## Materials and Methods

### Bacterial Strains and Growth Conditions

*Mycobacterium tuberculosis* clinical strains belonging to the Beijing family (Bisson et al., 2012) were obtained from the same patient before (parental, drug-susceptible strain) and after several weeks of anti-TB treatment (rifampin-resistant strain), respectively, and provided by Dr. Karen Dobos, Colorado State University. These two strains were used for all phenotypic studies. A wild-type Mtb CDC1551 and its lab-derived RpoB mutants H526D (Rifat et al., 2017) and D516V were also used to confirm our findings only in the vancomycin susceptibility assay.

Nutrient-rich conditions were established in Middlebrook 7H9 broth (Difco, Sparks, MD, United States) supplemented with 10% oleic acid-albumin-dextrose-catalase (OADC) (Difco), 0.1% glycerol, and 0.05% Tween-80 at 37°C on a shaker. For nutrient starvation experiments, bacterial pellets were washed three times with 1xPBS (Biological Quality) containing 0.05% Tween-80 and re-suspended in 10 ml of the same medium (at OD_600_ ∼ 0.1) in 50-ml conical tubes prior to standing incubation at 37°C for 14 days (Rifat et al., 2017).

### Whole Genome Sequencing

The paired clinical strains were sequenced on an Illumina HiSeq 2500 instrument using a paired-end (PE) sequencing strategy. DNA samples were extracted from colonies using the QIAamp DNA Mini Kit, sheared into ∼250 bp fragments using a Covaris sonicator (Covaris, Inc.), and prepared using the standard whole-genome DNA sequencing sample preparation kit (Illumina, Inc.). Paired-end reads of length 125 bp were collected. Base-calling was performed using HCS 2.2.58 and RTA 1.18.64 software (Illumina, Inc.). Based on spoligotype (000000000003771) and lineage-specific markers (GyrA:S95T, KatG:R463L), the strain was recognized to belong to the Beijing strain family. Reads were aligned to the genome of *M. tuberculosis* HN878 (representative Beijing strain; GenBank accession number ADNF01000000) as a reference sequence using BWA (Li and Durbin, 2009). Insertions and deletions were identified by local contig-building, as described previously (Ioerger et al., 2010). Polymorphisms (SNPs and indels) were identified as differences between the genomes of the two clinical isolates, excluding sites that were heterogenous (>30% non-majority nucleotides), low coverage (<10 reads), or in repetitive regions of the genome. Mean depth of coverage over the genomes was 53-fold.

### Transmission Electron Microscopy

Transmission electron microscopy (TEM) was performed as previously described (Chuang et al., 2015), with some modifications. Briefly, mid-log-phase bacteria growing in supplemented Middlebrook 7H9 broth or 14-day nutrient-starved bacteria were fixed with 3% formaldehyde, 2.0% glutaraldehyde, 80 mM Sorenson’s phosphate, and 4 mM MgCl2, pH 7.2. One half of the sample volume was processed for negative staining and the other for sectioning.

For negative staining, pellets in fixative were re-suspended and adsorbed to glow-discharged carbon-coated 400 mesh copper grids (EMS) for 2 min. Samples were stained after three quick TBS rinses, with 0.5% uranyl acetate containing 0.04% tylose as a wetting agent. Samples were blot-dried and stored in grid boxes until imaging. Samples with low bacterial numbers were spun down at 3,000 RPM onto grids in a Sorvall centrifuge for 5 min at 4°C.

For TEM sectioning, after the samples were rinsed three times with the same buffer containing 3.5% sucrose, they were post-fixed in 1% osmium tetroxide reduced with 0.8% potassium ferrocyanide in the same buffer without sucrose. After a brief water rinse, samples were stained en-bloc with filtered 2% uranyl acetate (aq.) for 1 h, then dehydrated through a graded series of ethanol to 100%, transferred to propylene oxide, and gradually infiltrated with a mixture of Spurr’s resin (Polysciences) containing increasing concentrations of propylene oxide (25, 50, and 75%) and rocked overnight. After three changes in pure Spurr’s resin, pellets were cured in a 60°C oven for 2 days. Sections were cut on a Reichert Ultracut E ultramicrotome with a Diatome diamond knife. Seventy-nanometer-thick sections were picked up on Formvar-coated 1- by 2-mm copper slot grids and stained first with 1% tannic acid (filtered aqueous), followed by 2% uranyl acetate (filtered aqueous), and then lead citrate. Grids were viewed on a Phillips CM 120 TEM operating at 80 kV, and digital images were captured with an XR-80 (8 megapixel) charged-coupled-device (CCD) camera.

### MIC and MBC Determination

The minimum inhibitory concentration (MIC) was determined as previously described (Chuang et al., 2013). Briefly, logarithmically growing Mtb strains (5 × 10^4^/ml) were inoculated in 15-ml conical tubes containing 2 ml of supplemented Middlebrook 7H9 broth without Tween-80 and with twofold increasing concentrations of antibiotics. Bacterial growth was determined by the presence of visible pellets after 14 days of standing culture at 37°C. The MIC was recorded as the lowest concentration of antibiotic for which there was no visible pellet. The minimum bactericidal concentration (MBC) was defined as the lowest concentration of antibiotic that yielded <1% survival of the initial inoculum. Briefly, the MBC was determined by transferring 100 μl from each tube containing no visible bacterial pellet and plating on Middlebrook 7H10 agar after serial dilutions. The plates were incubated at 37°C for 28 days prior to colony counting.

### Cell Wall Stress Assays

Heat shock, acid and sodium dodecyl sulfate (SDS) challenge were performed as previously described (Manganelli et al., 2001) with some modifications. Briefly, Mtb strains were grown to early log phase (OD_600_ = 0.4–0.8) in supplemented 7H9 Middlebrook broth. One aliquot of the culture was incubated in a water bath at 42°C for 24 h. Another aliquot was pelleted and resuspended in supplemented 7H9 Middlebrook broth pH 4.5 at an OD_600_ of 0.1 and incubated for 7 days at 37°C. SDS (0.05%) was added to a third aliquot, in which the bacterial inoculum was diluted to OD_600_ of 0.02, and incubated at 37°C for 6 h. The initial inocula were plated on Middlebrook 7H10 agar to determine the number of viable cells prior to stress exposure. At various predetermined time points, samples were diluted in PBS and plated to determine the CFU/ml. Results are expressed as the percentage of viable bacteria with respect to the initial inoculum. The same procedures were followed to determine the susceptibility of nutrient-starved bacteria to heat shock and SDS, except the initial inoculum was OD_600_ = 0.1. All experiments were performed using three biological replicates and were repeated at least twice.

### Redox Potential Assays

The resazurin assay was performed as previously described (Chuang et al., 2015). Briefly, serial dilutions of mid-log-growth-phase or nutrient-starved Mtb cultures were incubated with AlamarBlue (Invitrogen) for 18 to 20 h, and the fluorescence intensity was read by a BMG Optima microplate reader at 544-nm excitation and 590-nm emission wavelengths. The fluorescence intensity was normalized to bacterial density. Results yielding a linear relationship were used as representative data. All experiments were performed in triplicate and repeated at least twice, and similar results were obtained.

### Cell Wall Permeability Assays

Ethidium bromide (EthBr) accumulation/efflux and Nile red uptake were measured by fluorescence intensity, as previously described (Chuang et al., 2015) with minor modifications. Briefly, mid-log-phase or nutrient-starved cultures were washed with PBS and then stained with 2 μg/ml EthBr (Sigma) or with 0.125 μM Nile red (Sigma). For the EthBr accumulation assay with efflux inhibitor, verapamil (Sigma) was added and bacteria incubated with 2 μg/ml EthBr and 100 μg/ml verapamil for 60 min. In all assays, the cells were incubated in 96-well plates, and analysis was performed at the indicated time points by excitation at 544 nm and emission at 590 nm on a FLUOstar OPTIMA microplate reader (BMG LABTECH). All data were normalized to the time zero reading of each well and to bacterial density. All experiments were performed in triplicate and repeated at least twice, yielding similar results.

### Biofilm Formation Assays

Biofilms were assessed using Crystal violet staining, as previously described (Chuang et al., 2015) with minor modifications. Briefly, mid-log-phase cultures were diluted to a density of 10^6^/ml in Sauton’s medium without tween-80 and grown in 24-well plates at 37°C for 5 weeks. The extracellular biofilm matrix was measured using Crystal violet stain and a FLUOstar OPTIMA microplate reader (BMG LABTECH). All assays were performed in triplicate and repeated at least twice, yielding similar results.

### Statistical Analysis

Data from at least three biological replicates were used to calculate means and standard deviation (SD) for graphing purposes. Statistical analysis was performed by the unpaired Student *t*-test, and a *p*-value of <0.05 was considered significant. Analyses were performed using Prism 7 (GraphPad Software, San Diego, CA, United States).

## Results

### Whole Genome Sequencing of Mtb Strains

To characterize the genetic backgrounds of the paired clinical isolates recovered from the same patient with pulmonary tuberculosis before and after anti-TB treatment (Bisson et al., 2012), we performed whole genome sequencing. Sequencing analysis of genomic DNA from the rifampin-resistant post-treatment clinical strain revealed a C→G mutation at nucleotide position 1333 of the *rpoB* gene, causing an amino acid substitution of aspartate (D) for histidine (H) at codon 445 in the Mtb *rpoB* gene (hereafter referred to as RpoB mutation H526D, according to the *E. coli* annotation) and no additional mutations. Sequencing analysis of genomic DNA from the drug-susceptible clinical isolate revealed a G–A substitution 183 bp upstream of *rv1134*, a C–T substitution 10 bp upstream of *rv3303c/lpdA*, and a synonymous mutation in *rv3536c:v193V*, which were not present in the RpoB H526D mutant strain. However, these three sites exhibited heterogeneity in the sequencing data (mixture of nucleotides, from 76 to 91% mutated).

### RpoB Mutation H526D Is Associated With Slow Growth, Altered Mtb Colony Morphology, Bacillary Length and Cell Wall Thickness

**FIGURE 1:**
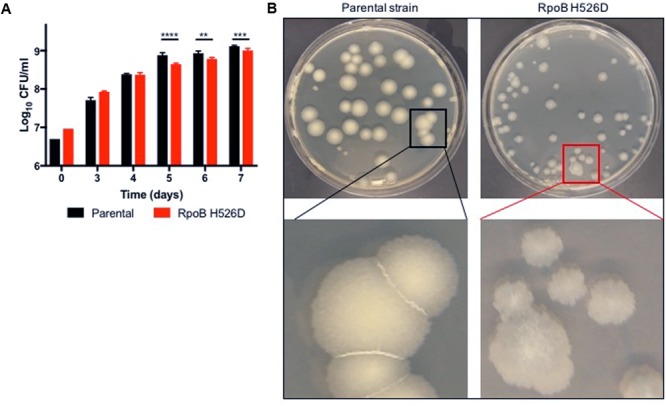
The *Mycobacterium tuberculosis* (Mtb) RpoB H526D mutant strain exhibits slow growth rate and altered colony morphology. **(A)** Growth kinetics of Mtb strains in supplemented Middlebrook 7H9 broth. ^∗∗^*p* < 0.01, ^∗∗∗^*p* < 0.001, ^∗∗∗∗^*p* < 0.0001. **(B)** Mtb cultures were grown on Middlebrook 7H10 agar and colonies were photographed 32 days post-inoculation. Colonies of the RpoB H526D mutant are smaller, more translucent, and less regular than those of the parental strain.

We have demonstrated previously that a laboratory-derived Mtb RpoB H526D mutant had a statistically insignificant fitness cost in nutrient-rich broth, but it was associated with small colony size after exposure to stress conditions (Rifat et al., 2017). Our clinically isolated Mtb RpoB H526D mutant showed a more profound phenotype, with a detectable effect on fitness, particularly after entering stationary phase (**Figure 1A**) and small colony size not only after exposure to stress conditions but also during growth in nutrient-rich conditions. Moreover, the RpoB H526D mutant colonies were more translucent and less regular than those of the rifampin-susceptible parental strain (**Figure 1B**). These colony phenotypes did not appear to be related to the smaller colony size of the RpoB H526D mutant strain since, with longer incubation times, the mutant colonies increased in diameter, attaining equivalent sizes to those of the parental strain, but retained the distinct morphological features (data not shown).

**FIGURE 2:**
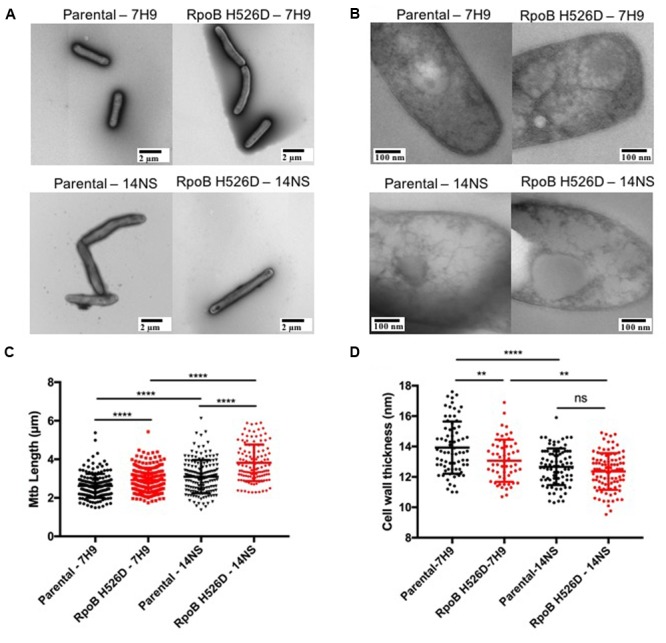
*Mycobacterium tuberculosis* RpoB mutation H526D is associated with altered bacillary length and cell wall thickness. The bacillary length **(A)** and cell wall thickness **(B)** of the RpoB H526D mutant and its parental strain were evaluated by transmission electron microscopy (TEM) during mid-log-growth-phase (7H9) and after exposure to nutrient starvation for 14 days (14NS). Bars, **(A)** 2 μm and **(B)** 100 nm. Dot plot graphs of **(C)** bacillary length (μm) and **(D)** cell wall thickness (nm). Each dot represents a measurement for a single bacillus. ^∗∗∗∗^*p* < 0.0001, ^∗∗^*p* < 0.01, ns: non-significant.

To further investigate these phenotypes, we studied bacillary morphology using TEM. During logarithmic growth in nutrient-rich broth, the mean length of RpoB H526D mutant bacilli along the longitudinal axis was significantly greater than that of the parental strain [2.92 ± 0.64 and 2.62 ± 0.62 μm, respectively (*p* < 0.0001)]. Following nutrient starvation for 14 days, the mean length of both strains increased significantly compared to their length in nutrient-rich conditions (**Figures 2A,C**). Interestingly, when comparing the mean length difference between the bacilli grown in nutrient-rich broth to that of the bacilli exposed to nutrient starvation for 14 days, we observed that this difference was significantly different among the mutant and the parental strain (*p* = 0.0002), since the mean length of the RpoB H526D mutant increased by 0.89 ± 0.94 μm after exposure to nutrient starvation, while that of the parental strain increased by only 0.47 ± 0.86 μm.

Additionally, we evaluated whether the modifications in the bacterial length were associated with changes in the cell wall thickness by TEM. Under nutrient-rich conditions, the mean cell wall thickness of the *rpoB* mutant (13.07 nm) was significantly (*p* = 0.0025) less than that of the parental strain (13.93 nm). Conversely, although the mean cell wall thickness of both strains decreased significantly after exposure to nutrient starvation for 14 days compared to that in nutrient-rich broth (**Figure 2B**), the change in cell wall thickness was significantly (*p* = 0.0024) greater in the parental (1.2 ± 1.2 nm) than in the *rpoB* mutant strain (0.7 ± 1.2 nm). Therefore, the mean cell wall thickness of the parental and *rpoB* mutant strains after exposure to nutrient starvation for 14 days was not significantly different (12.67 and 12.36 nm, respectively; **Figures 2B,D**).

### RpoB Mutation H526D Is Associated With Increased Mtb Cell Wall Permeability After Exposure to Nutrient Starvation

**FIGURE 3:**
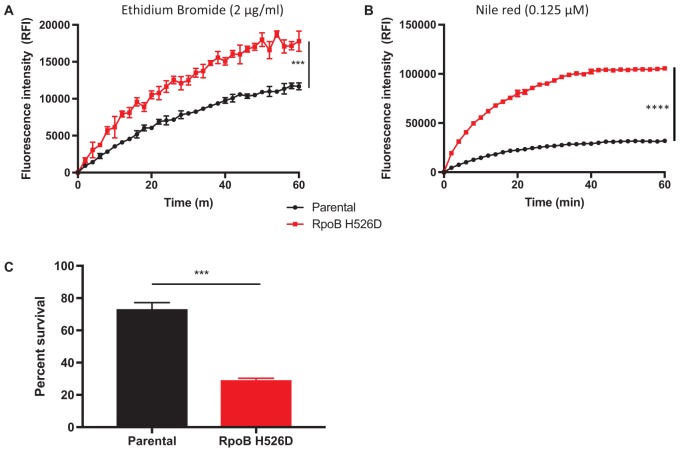
RpoB mutation H526D is associated with altered cell wall permeability and resistance to cell wall stress following nutrient starvation. Intracellular accumulation of ethidium bromide 2 μg/ml **(A)** and Nile red 0.125 μM **(B)** by the 14-day-nutrient-starved Mtb RpoB H526D mutant and its parental strain were measured by fluorescence spectroscopy. The values at each time point are normalized to the baseline fluorescence value and to bacterial density. RFI, relative fluorescence intensity. **(C)** Susceptibility of 14-day-nutrient-starved RpoB H526D mutant and parental strain to incubation with 0.05% sodium dodecyl sulfate (SDS) for 6 h. Percent survival represents the number of surviving bacteria after challenge divided by the number of bacteria prior to incubation. ^∗∗∗^*p* < 0.001, ^∗∗∗∗^*p* < 0.0001.

To determine whether the changes in bacillary cell wall physical properties observed in the RpoB H526D mutant were associated with altered cell wall permeability, we evaluated the rate of accumulation of the polar compound EthBr and the uptake of the lipophilic dye Nile red in the *rpoB* mutant and parental strains by fluorescence. EthBr is an intercalating agent that fluoresces only when bound to DNA. The lipophilic dye Nile red remains mainly periplasmic and binds to membrane phospholipids, where it fluoresces more strongly than when in aqueous solution. Therefore, both compounds are more fluorescent when located intracellularly rather than extracellularly and have been used to assess bacterial cell wall permeability (Blair and Piddock, 2016). Neither EthBr nor Nile red showed significantly different degrees of uptake or accumulation in the *rpoB* mutant compared to the parental strain during growth in nutrient-rich conditions (data not shown). However, following nutrient starvation for 14 days, the accumulation rate of EthBr was significantly higher in the *rpoB* mutant than in the parental strain (*p* = 0.0002; **Figure 3A**). The increased accumulation of EthBr in the nutrient-starved RpoB H526D mutant strain did not appear to be related to altered efflux as pre-incubation of each strain with the efflux pump inhibitor verapamil led to a similar increase in EthBr accumulation in each strain (data not shown). In addition, the RpoB H526D mutant showed higher uptake of Nile red relative to the parental strain (*p* < 0.0001) after nutrient starvation for 14 days (**Figure 3B**).

### RpoB Mutation H526D Leads to Increased Susceptibility of Nutrient-Starved Mtb to Cell Wall-Related Stress

Due to the altered cell wall permeability observed in the nutrient-starved RpoB H526D mutant, we evaluated the ability of the *rpoB* mutant to survive after exposure to the cell wall-perturbing detergent SDS, and during heat shock and acid stress. During growth in nutrient-rich broth, there was a rapid and similar decrease in bacterial viability for all tested strains exposed to SDS (data not shown). Following 14 days of nutrient starvation, the *rpoB* mutant was significantly less tolerant to SDS stress than the parental strain (*p* = 0.0004). After 6 h of exposure to SDS, the mean percent survival of the RpoB H526D mutant was 29%, while that of the parental strain was 73% (**Figure 3C**).

The susceptibility of the RpoB H526D mutant to heat shock (42°C) and acid stress (pH 4.5) was not significantly altered relative to the parental strain (data not shown).

### RpoB Mutation H526D Alters Bacterial Cellular Metabolic Activity But Not Biofilm Formation

**FIGURE 4:**
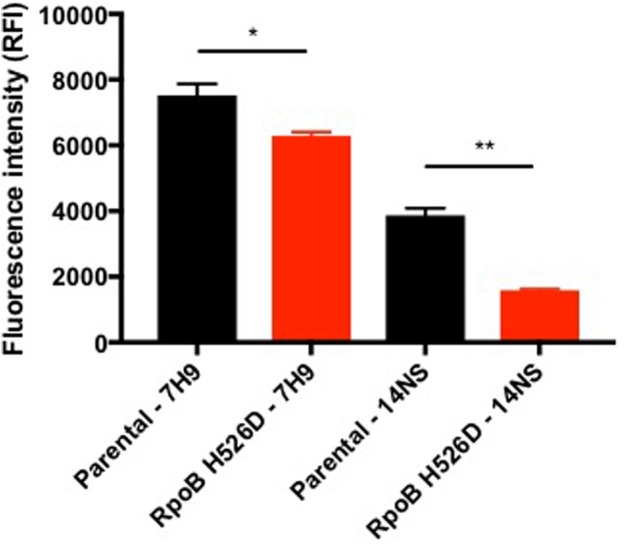
RpoB mutation H526D alters bacterial cellular metabolic activity. The RpoB H526D mutant and parental strains were grown to mid-log-phase in nutrient-rich broth (7H9) or were starved of nutrients for 14 days (14NS) prior to incubation with AlamarBlue for 18 h. The fluorescence signal was normalized based on bacterial density. RFI, relative fluorescence intensity. ^∗^*p* < 0.05, ^∗∗^*p* < 0.01.

The resazurin assay measures the ability of viable metabolically active cells to chemically reduce the substrate to the fluorescent molecule resorufin, which can be used as an indicator of cellular redox state and metabolic function (Rampersad, 2012; Chuang et al., 2015). In this assay, the *rpoB* mutant showed significantly decreased fluorescence intensity as compared to the parental strain in both nutrient-rich (*p* = 0.0296) and in nutrient starvation conditions (*p* = 0.0279) after exposure to resazurin for 18 h. In each condition, the normalized fluorescence signal (based on bacterial density) in the RpoB H526D mutant was 70% lower than that of the parental strain, consistent with reduced redox potential in the mutant (**Figure 4**).

Because the *rpoB* mutant strain showed increased cell wall permeability, susceptibility to cell membrane stresses and changes in cell wall thickness, we next sought to determine whether this mutation alters biofilm formation. The RpoB H526D mutant and parental strains were grown for 5 weeks in Sauton’s medium without detergent. We found that there were no significant differences between the quantity of biofilm formed by these strains as measured by Crystal violet staining (data not shown).

### RpoB Mutation H526D Is Associated With Increased Mtb Susceptibility to Vancomycin

The MIC of rifampin was determined to be >64 μg/ml against the RpoB H526D mutant and 0.125 μg/ml against the parental strain, while that of isoniazid was 0.025 μg/ml against each strain.

**FIGURE 5:**
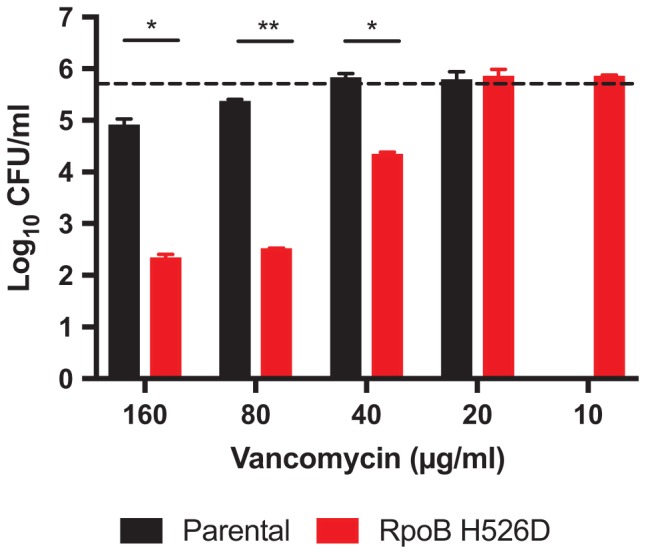
RpoB mutation H526D is associated with increased Mtb susceptibility to vancomycin. Bactericidal activity of vancomycin against RpoB H526D mutant and parental strains. The dashed line indicates the cell number for each strain at the start of the experiment (5.75 ± 0.01 log_10_ CFU/ml). ^∗^*p* < 0.05, ^∗∗^*p* < 0.01.

Intrinsic resistance of Mtb to beta-lactams and glycopeptides is thought to be due to reduced mycobacterial cell wall permeability. Thus, we evaluated whether the increased cell wall permeability observed in the RpoB H526D clinical isolate was associated with increased susceptibility to these antibiotics. Relative to the parental strain, the RpoB H526D mutant did not show altered susceptibility to the two beta-lactam antibiotics tested, meropenem and doripenem (MIC 8 and 4 μg/ml, respectively, against each strain). However, the MIC of vancomycin against the RpoB H526D mutant was eight times lower than that against the parental strain (**Table 1**). Measurement of bactericidal activity showed a similar tendency, as the MBC_99_ of the RpoB mutant was between 40 and 80 μg/ml whereas that of the parental strain was higher than 160 μg/ml, the maximum concentration tested (**Figure 5**). Similar results were obtained with Mtb CDC1551 lab-derived RpoB mutants H526D and D516V (**Table 1**).

**Table 1:** Vancomycin minimum inhibitory concentration (MIC) (μg/ml).

Parental	80
RpoB H526D	10
CDC1551	40
CDC1551 RpoB H526D	5
CDC1551 RpoB D516V	5

## Discussion

*Mycobacterium tuberculosis* resistance to environmental stress, antibiotics and therapeutic agents is due in part to the low permeability of its cell wall (Kieser and Rubin, 2014). The structure of the cell wall involves many unique lipids and glycolipids, deficiency of which has been associated with increased cell wall permeability and other phenotypes (Liu et al., 1996; Yuan et al., 1998; Camacho et al., 2001; Gebhardt et al., 2007; Gilmore et al., 2012; Reddy et al., 2013). Mutations in *rpoB* can potentially alter cellular function through alteration of Mtb transcriptional responses and, therefore, modify the structure, composition and/or integrity of the cell wall. Mtb *rpoB* mutant strains have altered expression of several cell wall proteins and lipids (Bisson et al., 2012; du Preez and Loots du, 2012; Lahiri et al., 2016). However, their effect on Mtb physiology and cell wall metabolism has not been well studied, especially during nutrient starvation, an *in vitro* stress condition that appears to elicit the persistent, antibiotic-tolerant state characteristic of *in vivo* bacteria (Lavollay et al., 2008). To our knowledge, our study is the first to show that the commonly observed RpoB mutation H526D is associated with increased cell wall permeability and decreased resistance to *in vitro* cell wall stresses.

Consistent with previous studies (Gagneux et al., 2006), the RpoB H526D mutant strain used in this study showed a statistically significant fitness cost in nutrient-rich broth. This mutation also had an effect on bacterial growth on Middlebrook 7H10 agar with small colony size and altered colony morphology. An altered mycobacterial colony phenotype has been associated previously with decreased expression of several Mtb molecules affecting bacterial cell wall composition and structure, including mycobactin, RpfAB and PDIM (Hotter et al., 2005; Russell-Goldman et al., 2008; Yu et al., 2012; Reddy et al., 2013). Although the cell wall permeability of the Mtb RpoB H526D mutant was not altered significantly during growth in nutrient-rich conditions, nutrient starvation significantly increased the cell wall permeability of this strain, which was not associated with decreased cell-wall thickness. Starvation conditions are known to cause downregulation of the biosynthesis of cell wall lipids, including PDIM, mycolic acids and mycobactin (Betts et al., 2002). Many of these cell wall molecules are already expressed at lower levels in *rpoB* mutants grown in nutrient-rich conditions (du Preez and Loots du, 2012; Lahiri et al., 2016). The expression of cell wall lipids may be further decreased in the nutrient-starved RpoB H526D mutant, compromising the overall integrity and permeability of the Mtb cell envelope. Transport of molecules through the cell wall can also be affected by efflux pump function. Although EthBr is a substrate for the efflux pump P55 (Farrow and Rubin, 2008), inhibition of efflux pump activity by the addition of verapamil did not differentially alter EthBr accumulation in the RpoB H526D mutant relative to the parental strain, suggesting that altered efflux pump activity does not play a role in the increased accumulation of EthBr in the nutrient-starved RpoB mutant strain.

The RpoB H526D mutant was more susceptible to the detergent SDS than the parental strain after exposure to nutrient starvation. Increased susceptibility to SDS has been associated with the absence of some cell wall lipids, including PDIM (Camacho et al., 2001), consistent with previous studies showing altered levels of PDIM precursors in various Mtb *rpoB* mutants (Bisson et al., 2012; Lahiri et al., 2016). This observation requires further analysis as it is possible that the increased expression of PDIM precursors is due to a feedback mechanism secondary to decreased levels of PDIM itself. Deficiency of the resuscitation-promoting factors RpfB and RpfE may also induce hypersensitivity to SDS (Kana et al., 2008). We have previously demonstrated that RpoB mutation H526D is associated with reduced expression of the genes *rpfB, rpfC*, and *rpfE* during resuscitation from growth-limiting conditions (Rifat et al., 2017). Therefore, reduced expression of these proteins may be contributing to the increased susceptibility of the RpoB H526D mutant to SDS.

RpoB mutation H526D also appeared to decrease the redox potential of Mtb under nutrient-rich and nutrient starvation conditions. This finding may be explained by the observation that *rpoB* mutations can mimic the stringent response (Koch et al., 2014), which is activated in response to nutrient starvation, thus enabling Mtb to restrict growth and shut down metabolism in a coordinated manner (Klinkenberg et al., 2010). The ability of *rpoB* mutants to simulate the stringent response may be beneficial for growth and survival in nutrient-limited environments (Koch et al., 2014) and for resistance to antimicrobial peptides (Gao et al., 2013). The synthesis of (p)ppGpp by Rel_Mtb_ during the Mtb stringent response regulates cell size and morphogenesis and therefore, may also play a role in the increased bacillary length observed in the RpoB mutant H526D, which has been previously described in MDR TB clinical isolates (Vijay et al., 2017).

The slowed growth rate of the RpoB H526D mutant in nutrient-rich broth did not altered its susceptibility to isoniazid as measured by MIC. This result is perhaps not surprising since the greatest anti-tubercular activity of isoniazid occurs within the first 2 days of antibiotic exposure (Jindani et al., 1980) and the growth phenotype of the *rpoB* mutant is not apparent until after 5 days of culture. In addition, a recent study showed equivalent bactericidal activity of isoniazid against slow-growing and rapidly growing cultures of Mtb during the first several generation times (Jeeves et al., 2015). Therefore, in our assays, isoniazid is expected to have retained most of its potency against the RpoB H526D mutant. The RpoB H526D mutant strain did not show altered susceptibility to meropenem or doripenem. However, the MIC of vancomycin against this strain was eightfold lower than that against the parental strain. A similar antibiotic susceptibility phenotype was observed in an Mtb strain lacking L,D-transpeptidases 1 and 2 (Schoonmaker et al., 2014). Mtb possesses L,D- and D,D-transpeptidases, both of which may be inhibited by carbapenems (Schoonmaker et al., 2014). It is possible that the RpoB H526D mutant has a lower expression of L,D-transpeptidases, but not of other transpeptidases, conserving a target for carbapenems. However, as L,D-transpeptidases act at a more distal step in peptidoglycan synthesis than vancomycin, a reduced level of these enzymes may slightly increase the abundance of target for vancomycin, increasing susceptibility to this drug. Depletion of RpfA and RpfB in Mtb strains has also been associated with increased susceptibility to vancomycin, perhaps by altering cell wall stability (Kana et al., 2010). Vancomycin itself can also alter cell wall permeability (Watanakunakorn, 1984). Therefore, decreased expression of Rpfs in the RpoB H526D mutant (Rifat et al., 2017) may enhance this mechanism of action of vancomycin. In addition, deletion of the *Rv1410c* gene, which encodes the P55 efflux pump, enhanced the susceptibility of *M. bovis* BCG to vancomycin, likely by altering proper cell wall assembly (Ramon-Garcia et al., 2009). Deficiency of different classes of Mtb efflux pumps (Rv0849, Rv1218c, Rv1258c, and Rv3065) also increased susceptibility to vancomycin but not to meropenem (Dinesh et al., 2013). However, the expression of Rv1410c and Rv0849 was found to be significantly higher in MDR-TB strains as compared to pan-susceptible strains (Li et al., 2015), suggesting they do not play a role in the increased susceptibility to vancomycin observed in the RpoB H526D mutant strain. Lack of PDIM in Mtb mutants has also been associated with increased susceptibility to vancomycin (Soetaert et al., 2015; Rens et al., 2016). Determining whether PDIM content is altered in Mtb *rpoB* mutants can provide some insight into the contribution of this molecule to their altered susceptibility to vancomycin.

Although genetic complementation is often used to confirm that particular bacterial phenotypes are due to the mutation of interest, our prior work showed that complementation of a laboratory-derived Mtb RpoB H526D mutant with a wild-type copy of the *rpoB* gene led to incomplete restoration of the wild-type phenotype, likely due to increased expression of the *rpoB* gene in the merodiploid complemented strain (Rifat et al., 2017). We are highly confident that the mutant phenotypes we observed in the current study are attributable to the RpoB H526D mutation since whole-genome sequencing revealed only this mutation in the rifampin-resistant mutant, and a lab-derived rifampin-resistant strain containing the same mutation exhibited similar phenotypes. Interestingly, we detected three additional mutations in the drug-susceptible parent strain, which was isolated from the same patient at an earlier time relative to the rifampin-resistant strain. As mentioned above, the sequencing data showed heterogeneity at these three sites, suggesting the possibility that the pre-treatment parent isolate might have been mixed, with DNA from several co-existing clones *in vivo*, some of which might have acquired additional mutations during the course of infection (or from a secondary infection). We favor this explanation as being statistically more likely than the alternative one, i.e., reversion of three different SNPs in the indicated genes, although confirmation would require sequencing of 10–20 distinct colonies from the drug-susceptible parent strain to be able to identify whether some members of the population had the wild-type nucleotide in genes *rv1134* and *rv3563c*, given the heterogeneity of 10 and 9%, respectively, detected by sequencing at each of these sites. In the scenario we have proposed, one of the original clones lacking these mutations ultimately acquired the *rpoB* mutation and was selected to become the dominant clone during anti-TB treatment with rifampin.

Taken together, the results from our study indicate that Mtb RpoB mutation H526D alters cell wall physiology, increasing permeability and reducing Mtb resistance to cell wall-damaging agents, particularly after exposure to nutrient starvation, and increases susceptibility to the cell wall-acting drug vancomycin. These cell wall phenotypes might not be exclusive to RpoB mutation H526D and Mtb strains containing mutations in other RpoB codons might show similar cell wall-related modifications, as previous studies on the expression of cell wall lipid metabolites, as well as enzymes involved in PDIM synthesis, showed reproducible patterns in all Mtb *rpoB* mutants tested, regardless of the specific amino acid mutation (Bisson et al., 2012; du Preez and Loots du, 2012; Lahiri et al., 2016). Although it remains to be determined how Mtb bearing this epidemiologically common *rpoB* mutation overcomes its virulence defects in order to be transmitted and cause disease in the human host, we expect that the findings reported in this study will contribute to a better understanding of MDR TB pathogenesis.

## Data Availability Statement

The raw data supporting the conclusion of this manuscript will be made available by the authors, without undue reservation, to any qualified researcher.

## Author Contributions

VC and PK conceived the experiments, wrote and reviewed the article. VC, DR, Y-MC, and PK designed the experiments. VC conducted the experiments, data analysis and interpretation. TI performed the whole genome sequencing analysis and interpretation.

## Conflict of Interest Statement

The authors declare that the research was conducted in the absence of any commercial or financial relationships that could be construed as a potential conflict of interest.
